# A review of multiple diagnostic approaches in the undiagnosed diseases network to identify inherited metabolic diseases

**DOI:** 10.1186/s13023-024-03423-3

**Published:** 2024-11-14

**Authors:** Yutaka Furuta, Rory J. Tinker, Rizwan Hamid, Joy D. Cogan, Kimberly M. Ezell, Devin Oglesbee, Ralph J. DeBerardinis, John A. Phillips, the Undiagnosed Diseases Network

**Affiliations:** 1 Department of Pediatrics, Division of Medical Genetics and Genomic Medicine, Vanderbilt University Medical Center, 2200 Children’s Way, Nashville, TN 37232 USA; 2 Department of Laboratory Medicine and Pathology, Mayo Clinic, Rochester, MN USA; 3 Children’s Medical Center Research Institute, University of Texas Southwestern Medical Center, Dallas, TX USA; 4 Howard Hughes Medical Institute, University of Texas Southwestern Medical Center, Dallas, TX USA

**Keywords:** Inherited metabolic diseases (IMDs), Next generation sequencing (NGS), Undiagnosed Diseases Network (UDN)

## Abstract

**Background:**

The number of known inherited metabolic diseases (IMDs) has been expanding, and the rate of diagnosis is improving with the development of innovative approaches including next generation sequencing (NGS). However, a substantial proportion of IMDs remain undetected by traditional diagnostic approaches. We aim to highlight the spectrum of IMDs diagnosed by the Undiagnosed Diseases Network (UDN) and to learn from the UDN diagnostic processes that were able to detect IMDs.

**Methods:**

We conducted a retrospective analysis of 757 UDN participants diagnosed from 2015 until 2023 using the cohort database, which were divided into a cohort with IMDs (*n* = 194; 27%) and a cohort whose phenotypes were not explained by an IMD (*n* = 563; 73%), based on the International Classification of Inherited Metabolic Disorders (ICIMD). Then, we divided the causes of the metabolic 194 diagnoses into seven groups that included all the ICIMD categories. We inspected which clinical and laboratory approaches contributed to a final UDN diagnosis. We also present a UDN case example from each group to highlight the diagnostic yields that resulted from combining newer diagnostic approaches in the UDN and illustrate potential pitfalls of current NGS methods.

**Results:**

These 194 cases of IMDs included examples from 21/25 (84%) of the ICIMD categories. Of the UDN subjects 164/194 (85%) were diagnosed with IMDs through NGS.

**Conclusion:**

The spectrum of IMDs detected in the UDN cohort is large and growing and appropriate use of newer multiple diagnostic approaches should further increase diagnosis of IMDs that are presently missed by the traditional laboratory screening methods.

## Background

Inherited metabolic diseases (IMDs) are a large group of single-gene disorders resulting from enzyme defects in biochemical and metabolic pathways [1]. While each IMD is rare, they collectively affect ~ 1 in 780 births [2]. With the development and application of innovative diagnostic methods, insight to IMDs has progressively expanded. The latest and most comprehensive classification of IMDs, known as the International Classification of Inherited Metabolic Disorders (ICIMD) was updated in 2021 and includes ~ 1,450 IMDs [3].

Classic diagnostic methods to detect IMDs include newborn screening (NBS), tandem mass spectrometry, and biochemical studies (e.g., plasma amino acids, acylcarnitine profiles, urine organic acids). However, it is recognized that some IMDs lack reliable and accessible biochemical markers, which can limit approaches to diagnose IMDs through direct biomarker measurements [4]. Limitations include both physical and biochemical phenotypic signatures that can fluctuate depending on clinical status. Furthermore, Ghosh et al., suggest that the reliance on only “traditional” approaches contribute to a substantial proportion of IMDs remaining undiagnosed.

Currently, the detection of rare and undiagnosed genetic disorders is improved by next generation sequencing (NGS) methodologies, which includes exome (ES) and genome sequencing (GS). In the same vein, NGS has significant promise for clinical utility in diagnosing IMD. However, NGS has reduced sensitivity and availability as well as increased turn-around time and costs when compared to traditional approaches to IMDs which can pose barriers to the clinical use and diagnostic utility of NGS. In fact, Schuler et al., presented examples of limitations and failures of NGS to detect the genetic diseases which further illustrate the reduced sensitivity of NGS [5].

The Undiagnosed Diseases Network (UDN) is a research study funded by the National Institutes of Health (NIH), which aims to identify and diagnose undiagnosed diseases. To begin the UDN enrollment, patients submit an application and their referring clinicians submit a nominating letter to the “UDN Gateway,” which is managed by the Coordinating Center (https://undiagnosed.hms.harvard.edu/apply/). Once the application and nominating letter are submitted online, these along with requested medical records are routed to a Clinical Site, typically the one nearest to the applicants. The Clinical Site’s team review the applications along with medical records and nominating letter to determine whether to accept applicant and proceed with a UDN evaluation. In addition to the Coordinating Center and Clinical Sites, the UDN has a Sequencing Core, Model Organisms Screening Centers, Metabolomics Core, and Central Biorepository. The UDN does not dictate the use of specific software for data analysis, and each Site and Core may utilize different analytical platforms that they have found to be optimal. Goals of the UDN include to facilitate research on undiagnosed diseases and create a collaborative research community to identify improved options for patient care. To do this the UDN Data Management Coordinating Canter facilitates sharing of de-identified data with NIH-designated repositories and other relevant resources. Genomic and phenotypic data are made available to the scientific community through the dbGaP, with updates submitted annually. Researchers can access data on the UDN study page in dbGaP, but they must submit data requests to obtain access. Also, researchers who are interested in collaborating on UDN cases can contact the UDN at https://undiagnosed.hms.harvard.edu/apply/ to discuss their interests and request and they can also register on ModelMatcher.

In this study, we review the number of UDN participants who obtained final diagnosis of an IMD that required the use of newer diagnostic approaches, such as NGS. Importantly, prior to their acceptance by the UDN all participants were required to have had clinical and genetic evaluations that had typically included NBS, tandem mass spectrometry, biochemical studies, gene-panel sequencing, and occasionally, exome sequencing (ES).

We hypothesized that multiple diagnostic approaches including NGS would be required to detect IMDs. In addition, we also hypothesized that we would learn lessons about the possible benefits that arose from combining emerging diagnostic methods to improve the diagnostic yield of IMD among UDN participants. To determine the limitations of the traditional diagnostic approaches as well as the advantages and limitations of using newer methods including NGS in diagnosing IMDs, we conducted a retrospective analysis of the UDN cohort of diagnosed participants. We compared the diagnostic yield of different genetic tests in diagnosing IMDs in UDN participants.

## Materials and methods

The UDN is supported by NIH Common Fund, NIH/NHGRI grants. The UDN participants were consented to the study using IRB protocol, NHGRI 15-HG-0130 before any studies or evaluations were performed. We completed a retrospective analysis of the UDN cohort of diagnosed participants that included all 757 UDN participants diagnosed from 2015 until 2023. First, all 757 subjects were divided into a group with IMDs and a group whose phenotypes were not explained by an IMD, based on the International Classification of Inherited Metabolic Disorders (ICIMD) [3]. To consider whether each disease should be classified as an IMD, we utilized IEMbase (www.iembase.org) [6] and the ICIMD nosology number. We found that 194 participants were diagnosed with a condition included in the IMD classification. Second, we divided the causes of the 194 IMD diagnoses into 7 groups that included all the ICIMD categories [3]. Our groups included the following categories: group 1 contains ICIMD categories 1–4, group 2 categories 5–11, group 3 category 14, group 4 category 16, group 5 categories 18–20, group 6 categories 21–22, and group 7 categories 23–24 (See Table 1). There were no UDN cases that corresponded to ICIMD categories 12, 13, 15, or 17. We then determined which diagnostic approaches were used to diagnose each UDN participant to establish their IMD diagnosis.

**Table 1:** Our seven groups and the correspondence of ICIMD categories

Our groups	Group name	Correspondence of ICIMD categories (Ferreira et al., 2021)
1	Intermediary metabolism: Nutrients	1–4
2	Intermediary metabolism: Energy	5–11
3	Lipid metabolism and transport	14
4	Metabolisms of heterocyclic compounds	16
5	Complex molecule and organelle metabolism	18–20
6	Cofactor and mineral metabolism	21–22
7	Metabolic cell signaling	23–24

## Results

**Fig. 1:**
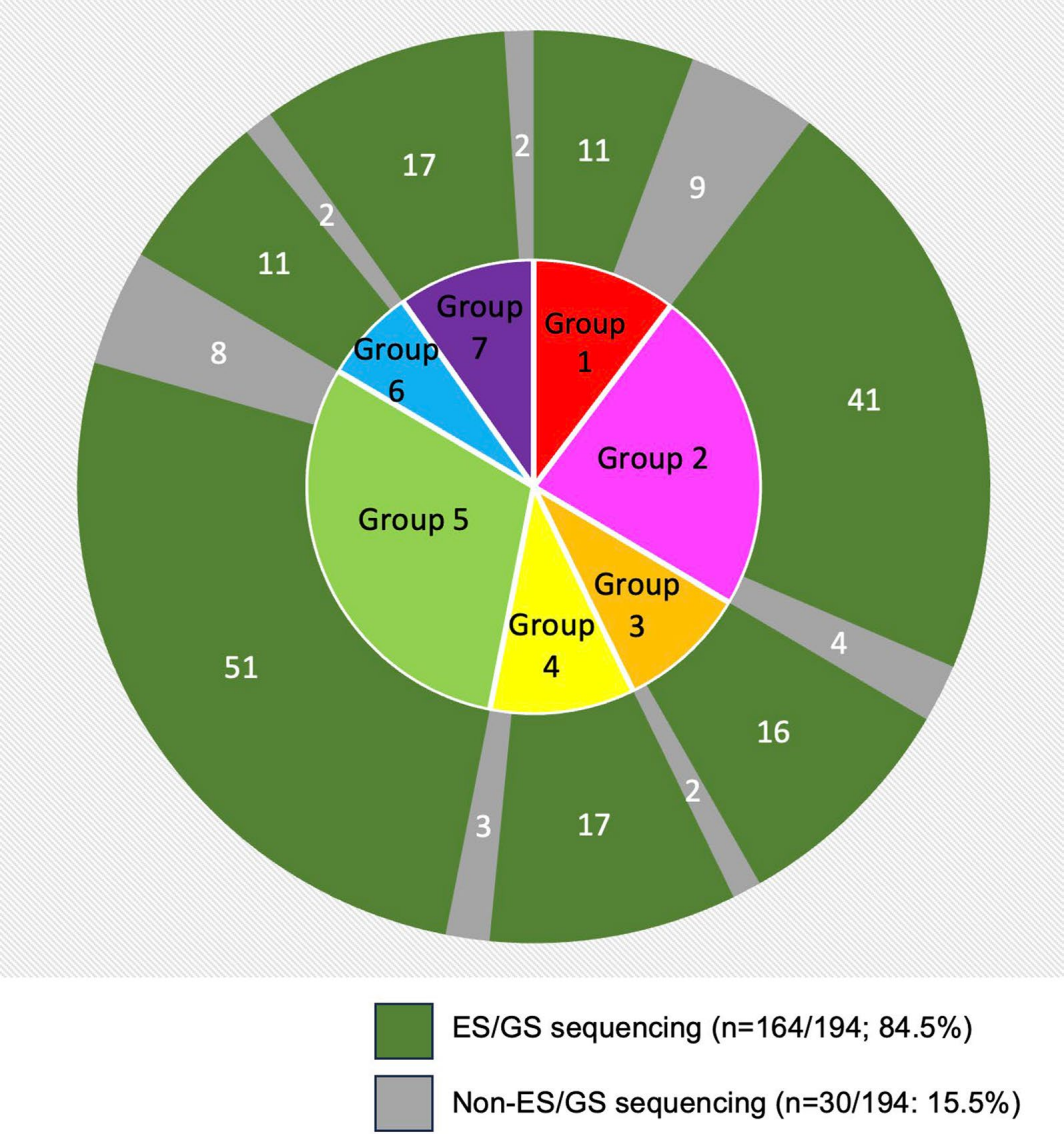
UDN subjects diagnosed as having inherited metabolic diseases by ES/GS sequencing versus non- ES/GS sequencing approaches

Among 757 UDN participants, there was a cohort in which phenotypes were explained by an IMD in 194/757 (27%), and a cohort in which phenotypes were not explained by an IMD in 563/757 (73%) but were diagnosed with an alternative non-metabolic disease. Of 194 UDN IMD cases, we found that 20/194 (10%), 45/194 (23%), 18/194 (9%), 20/194 (10%), 59/194 (31%), 13/194 (7%), and 19/194 (10%) were in groups 1–7, respectively. Of these 194 cases in groups 1–7 the mean number, median number, and standard deviations were 27.7, 20, and 15.9, respectively. In 164/194 cases (84.5%), Exome or genome sequencing (ES/GS) was required to make the final UDN diagnosis (see Fig. 1). Biochemical phenotyping was diagnostic in 3/20 (15%) and 2/45 (4%) of cases in groups 1 and 5, respectively. Clinical phenotyping was diagnostic in 3/20 (15%), 1/45 (2%), 1/20 (5%), 1/59 (2%), and 1/13 (8%) of cases in groups 1, 2, 4, 5, and 6, respectively. Chromosomal microarray (CMA) was diagnostic in 1/19 (5%) of cases in group 7. Further in *vitro/vivo* studies were needed to diagnose 1/59 (2%) of cases in group 5. Mitochondrial DNA sequencing was diagnostic in 2/45 (4%) of cases in group 2. Gene panels were diagnostic in 2/45 (4%) and 2/13 (15%) of cases in the groups 2 and 6, respectively. RNA sequencing contributed to the diagnoses of 3/20 (15%), 1/20 (5%), 1/59 (2%), and 1/19 (5%) of cases in the groups 1, 4, 5, and 7, respectively. Single gene tests were diagnostic in 2/18 (11%), 1/20 (5%), and 3/59 (5%) of cases groups 3, 4 and 5, respectively (See Table 2).

**Table 2:** Type of key diagnostic approach to inherited metabolic diseases in the UDN by categories

Group	Case (n)	Cases diagnosed by ES/GS sequencing	Cases diagnosed by biochemical phenotyping	Cases diagnosed by clinical phenotyping	Cases diagnosed by CMA	Cases diagnosed by vitro/vivo studies	Cases diagnosed by mtDNA sequencing	Cases diagnosed by gene panel	Cases diagnosed by RNA sequencing	Cases diagnosed by single gene tests
1 Intermediary metabolism: Nutrients	20	11	3	3	0	0	0	0	3	0
2 Intermediary metabolism: Energy	45	41	0	1	0	0	2	1	0	0
3 Lipid metabolism and transport	18	16	0	0	0	0	0	0	0	2
4 Metabolisms of heterocyclic compounds	20	17	0	1	0	0	0	0	1	1
5 Complex molecule and organelle metabolism	59	51	2	1	0	1	0	0	1	3
6 Cofactor and mineral metabolism	13	11	0	1	0	0	0	1	0	0
7 Metabolic cell signaling	19	17	0	0	1	0	0	0	1	0
Total	194	164	5	7	1	1	2	2	6	6

## Case series in the UDN cohort

In the following section, we present a case example of each group of IMDs to highlight the approach and diagnostic yields that resulted from traditional and combining newer diagnostic approaches in the UDN as well as the potential pitfalls of NGS (summarized in Table 3). These seven cases were selected from previously published UDN articles.

**Table 3:** UDN Case series

Case	Group	Disease/Gene	PMID	Key diagnostic approach in the UDN	Lesson learned
1	1. Intermediary metabolism: Nutrients	SORD deficiency / *SORD*	37,622,199	GS	GS may be beneficial in some individuals with unknown genetic disease not detected by ES GS reanalysis after a year can be beneficial because of subsequent discoveries
2	2. Intermediary metabolism: Energy	Complexes II and III deficiency / *SDHA*	33,960,148	ES followed by respiratory chain functional study	ES may be a useful diagnostic tool NGS can be “required but not sufficient” to confirm the diagnosis
3	3. Lipid metabolism and transport	Neurodegeneration with brain iron accumulation 2A/ *PLA2G6*	28,914,269	Sanger sequencing & copy number testing guided by phenotypic information	Phenotype-guided molecular testing can help in detecting candidate variants that might have been missed by ES done to avoid GS
4	4. Metabolisms of heterocyclic compounds	Shwachman-Diamond syndrome 2 / *EFL1*	29,970,384	Reanalysis of ES	Reanalysis of ES may help to avoid unnecessary GS
5	5. Complex molecule and organelle metabolism	GET4 deficiency / *GET4*	32,395,830	ES and GS	ES/GS are both useful tools with different sensitivities and expenses
6	6. Cofactor and mineral metabolism	NADK2 deficiency /* NADK2*	29,388,319	ES	ES is a useful diagnostic tool when the phenotype is not specific
7	7. Metabolic cell signaling	*CLTC*-related syndrome /*CLTC*	33,001,864	GS	GS may detect candidate variants missed by ES and/or CMA

***Group 1*:** A 72-year-old male was referred to the UDN because of gradual onset progressive weakness in both lower extremities for the past 45 years. The primary diagnostic consideration was Charcot-Marie-Tooth (CMT) disease type 2, but a CMT disease comprehensive panel was negative. Exome sequencing (ES) was negative for deleterious neuromuscular variants. He was referred to the UDN, where he underwent GS. His initial UDN GS report did not include his *SORD* variant, but his final UDN report included homozygosity for a *SORD* frameshift terminating variant (c.757delG:p.A253Qfs*27), which was masked by pseudogene, *SORD2P*, interference. In addition, his urine sorbitol level was significantly elevated, which confirmed SORD deficiency (OMIM #618,912). SORD deficiency is an autosomal recessive form of CMT type 2 characterized by a progressive peripheral neuropathy due to accumulation of intracellular toxic sorbitol. His variants in *SORD* are reported to be the most common cause of SORD deficiency and subsequently the most common cause of recessive neuropathy [7].

***Group 2***: A 9-year-old male was referred to the UDN with a history of tremor, nystagmus, hypotonia, developmental delay, ataxia, and progressive cerebellar atrophy. While his lysosomal enzyme studies showed mildly reduced activities of beta-galactosidase and beta-glucuronidase, other biochemical studies were normal including cerebral spinal fluid glucose, amino acids, neurotransmitter metabolites, tetrahydrobiopterin, neopterin, and 5-methyltetrahydrofolate levels. Mitochondrial genome sequencing was reported as normal. His UDN ES identified compound heterozygous variants in *SDHA* with a maternally inherited, c.91C > T (p.R31*), known pathogenic terminating variant and a paternally inherited, c.454G > A (p.E152K), variant of unknown significance (VUS). Then, mitochondrial respiratory chain enzyme function using skin fibroblasts was obtained that showed deficient activity of complexes II and III. His c.454G > A (p.E152K) *SDHA* variant which had been previously classified as a VUS, was reported to be pathogenic in an updated lab report. Mitochondrial Complex II deficiency, Nuclear Type 1 (OMIM #252,011) is an autosomal recessive disorder. The spectrum of clinical phenotypes of SDHA-related disease are quite broad, including optic atrophy, ocular movement disorders, neuropathy, progressive neuromuscular decline, ataxia, Leigh syndrome, and cardiomyopathy. The authors concluded that the lack of symptoms in the parents was expected because of the autosomal recessive mode of inheritance [8].

***Group 3***: A 3.5-year-old female was referred to the UDN with a history of developmental regression, cerebellar atrophy, optic atrophy, and profound generalized hypotonia. Her brain MRIs demonstrated white matter volume loss of the vermis and cerebellar hemispheres. Her clinical features were strongly thought to be due to infantile neuroaxonal dystrophy (IND). However, metabolic laboratory tests and an ataxia gene panel (42 genes) were negative. Trio ES through a commercial laboratory was also negative. She was referred to the UDN, where Sanger sequencing and a multiple ligation-dependent probe amplification (MLPA) study were done to detect deletions/duplications and other variants. Sanger sequencing results were reported as normal. However, her MLPA showed a novel homozygous deletion of the non-coding *PLA2G6* exon 1, which was not included in the ES capture kit. This result confirmed the clinical suspicion of neurodegeneration with brain iron accumulation 2A (NBIA2A) (OMIM #256,600). NBIA2A is an autosomal recessive neurodegenerative disease characterized by abnormal accumulation of iron in the basal ganglia, resulting in generalized cerebral and cerebellar atrophy, optic atrophy, progressive dystonia, dysarthria, spasticity, and parkinsonism [9].

***Group 4*:** A 14-year-old female was referred to the UDN with a history of metaphyseal dysplasia, thrombocytopenia, growth failure, liver fibrosis, scoliosis, and learning disabilities. Prior to the UDN entry, she had had extensive clinical and molecular evaluations including trio ES at age of 10 years, without a genetic diagnosis. However, subsequent reanalysis of her ES data through the UDN demonstrated homozygosity for a *EFL1* p.Thr127Ala missense pathogenic variant, which was thought to explain her Shwachman-Diamond syndrome-like phenotype (OMIM #260,400). The variant had not been initially reported because of the research laboratory focused only on de novo variants. Shwachman-Diamond syndrome is an autosomal recessive multisystem disorder characterized by skeletal abnormalities, especially bone metaphyseal dysostosis, bone marrow dysfunction, and exocrine pancreatic dysfunction. While her metaphyseal dysplasia was more severe than in the original reported cohort with *EFL1* variants, her bone marrow dysfunction was milder. Different *EFL1* variants can contribute to a broad spectrum of symptoms that overlap with those observed in Shwachman-Diamond syndrome [10].

***Group 5*:** A 10-year-old male was referred to the UDN with a history of global developmental delay, intellectual disabilities, uncontrolled seizures, facial dysmorphism, delayed bone age, scoliosis, brachycephaly, microcephaly, and agenesis of the corpus callosum on a brain MRI. His extensive biochemical genetics evaluations included a Carbohydrate Deficient Transferrin test, which revealed a modest type II glycosylation pattern lacking a single sialic acid. However, a DNA gene panel for disorders of glycosylation was reported as negative for relevant variants. His UDN evaluations included both ES and GS since his clinical lab testing failed to reveal genetic causes. Both ES and GS results demonstrated compound heterozygosity for p.Arg122His and p.Ile279Met *GET4* candidate variants and he was diagnosed as having GET4 deficiency (OMIM #612,056). He was reported as the first individual with GET4 deficiency causing congenital disorder of glycosylation type IIy. This is an autosomal recessive multisystemic disorder characterized by poor growth, global developmental delay, and intellectual disabilities, hypotonia, seizures, brain imaging abnormalities, dysmorphic features, and skeletal defects [11].

***Group 6*:** A 15-year-old female was referred to the UDN with a history of decreased visual acuity, optic atrophy, nystagmus, episodic lower extremity weakness with elevated creatine phosphokinase levels, peripheral neuropathy, and gait disturbance. Her previous testing had included biochemical studies. Her plasma amino acids (PAA) showed elevated lysine and proline levels. While her acylcarnitine profile (ACP) revealed elevations of C10 to C18, suggestive of mild multiple Acy-CoA-Dehydrogenase Deficiency (MADD), her other ACP values were normal. Her urine organic acids showed severe lactic aciduria, but no glutaric acid elevation, which made MADD less likely. Her free and total carnitine levels suggested mild secondary carnitine deficiency. Her vitamin B12 levels were decreased. Her UDN ES showed homozygosity for a *NADK2* p.Met1Val start loss pathogenic variant, which confirmed her diagnosis of NAD kinase deficiency (NADK2D, OMIM #615,787). NADK2D is a rare autosomal recessive disorder of NADPH biosynthesis that results in hyperlysinemia and dienoyl-CoA reductase deficiency (DECRD, OMIM #616,034). She was reported as the fourth patient with NADK2D [12].

***Group 7*:** A 14-year-old male was referred to the UDN with a history of global developmental delay, intellectual disabilities, dysmorphic features, and seizures. His genetic tests had included a karyotype, chromosomal microarray (CMA), trio (parental and subject) ES, and fragile X testing, which were all reported as negative. RNA sequencing revealed approximately half the normal amounts of *CLTC* and *PTRH2* transcripts. Since both genes are adjacent to each other on chromosome 17q23.1, these results suggested a possible contiguous deletion involving both genes. A UDN GS was performed that demonstrated a heterozygous *CLTC* deletion that removed the segment from exons 18–32, a transcription end of *CLTC* and part of the adjacent *PTRH2*. This partial *CLTC* deletion was missed on his trio ES, and it was not covered by his previous CMA. His *CLTC* deletion was confirmed by Polymerase chain reaction (PCR) analysis. He was diagnosed as having a *CLTC*-related syndrome (OMIM #617,854). This is an autosomal dominant intellectual developmental disorder with a broad spectrum of neurodevelopmental phenotypes, including developmental delay, intellectual disabilities, and epilepsy as well as nonspecific facial dysmorphisms. These features largely overlapped with his clinical presentations [13].

## Discussion

Overall, we found that 194/757 (27%) of UDN diagnoses were classified as IMDs, showing that IMDs comprise a considerable proportion of undiagnosed diseases. These 194 cases included examples from 21/25 (84%) of all ICIMD categories mentioned above apart from 12, 13, 15, and 17, indicating that UDN subjects have a broad range of IMDs.

We demonstrated in Table 2 that a wide variety of diagnostic methods were needed to evaluate and diagnose an IMD in UDN subjects, including ES/GS, biochemical phenotyping, clinical phenotyping, CMA, *vitro/vivo* studies, mitochondrial DNA sequencing, gene panels, single gene tests, and RNA sequencing. Interestingly, of the UDN subjects 164/194 (85%) were diagnosed as having IMD by ES/GS (see Fig. 1). While at least 85% of cases in groups 2–7 were diagnosed by ES/GS, 11/20 (55%) of cases in group 1 achieved a diagnosis with ES/GS. The remaining 9/20 (45%) in group 1 were diagnosed by biochemical phenotyping, clinical phenotyping, or RNA sequencing. This suggests that nearly half of amino acid, carbohydrate, and fatty acid metabolisms disorders are simple to diagnose with traditional diagnostic approaches and without ES/GS.

While 85% of UDN subjects achieved diagnoses of IMD through ES/GS, this is misleading because the UDN has a selection bias. To be nominated to the UDN, subjects should have objective, significant signs and symptoms, thorough clinical and laboratory evaluations. Thus, most have already had classical metabolic screening tests as part of their previous evaluations, and were nominated because these studies were nondiagnostic. As a result, accepted UDN subjects tend to have more complex and challenging diagnoses odysseys because easy diagnoses would have been made by traditional IMD tests. The formal criteria for acceptance to the UDN include 1) applicants should have one or more objective findings pertinent to their abnormal phenotype, 2) they were not diagnosed despite evaluations by pertinent specialists who assessed them for the objective findings, 3) a formal nomination letter is submitted that summarizes their medical and family history as well as their signs and symptoms and previous lab and other tests including CMA and DNA gene, panel or ES if clinically indicated. This complex selection process likely contributes to the high success rate of ES/GS in making UDN diagnoses. In addition, there is a bias resulting from the selection of diagnostic tool when evaluating UDN subjects that contributes to a high success rate for ES/GS among the UDN cohort. At the onset of the UDN study, ES/GS methods were frequently used within the UDN, but were less accessible to external patients, due to costs and insurance coverages. However, costs to UDN participants were minimized regardless of their insurance status, which enabled UDN subjects additional opportunities to receive more comprehensive UDN diagnostic evaluations including ES/GS.

While UDN ES had powerful diagnostic utility as described in Table 2 and seven cases (Table 3), it may miss diagnoses. In fact, 33% of the UDN cases were reported not to be solved exclusively by ES [14]. In our group 7 case [13], the *CLTC* deletion was detected by GS through the UDN, which was missed on trio ES and not covered by previous CMA. As Murdock et al., concluded, GS can be useful in identification of variants missed on ES and CMA. In our group 3 case [9], her deletion of the non-coding *PLA2G6* exon 1 was detected through Sanger sequencing and MLPA with phenotypic information while it was missed on Trio ES through a commercial laboratory. Pena et al., comment that GS was an impractical option given its limited availability and lack of validation although the variant should have been detected by GS. ES can have limitations due to procedures such as sequences captured, literature review as well as its poor ability to detect non-coding or copy number variants. In our group 1 case [7], an ES done prior to the UDN entry failed to detect deleterious neuromuscular variants. However, this subject was formally diagnosed as having SORD deficiency due to homozygous *SORD* (c.757delG:p.A253Qfs*27) frameshift mutation found through UDN GS. This case is a good example to highlight the utility of GS for a subject whose ES did not provide a diagnosis. Interestingly, the variant was also missed in the initial UDN GS report but was detected through the UDN GS reanalysis. This illustrates how reanalysis of NGS data can improve the diagnostic rate because of pipeline improvements and inclusion of subsequently discovered disease-causing alleles and genes that were not available at the time of the first analysis. In fact, 26–43% of UDN diagnoses have been found by NGS reanalysis [15, 16].

While GS is the most common diagnostic approach in the UDN in cases that are not solved by ES as described the above, GS is not always done to detect genetic causes that ES may miss. In our group 4 case [10], the genetic diagnosis was obtained by reanalysis of ES data through the UDN. In this case the initial ES analysis failed to detect the homozygous missense variant in *EFL1* and the option of re-evaluating the ES data instead of proceeding to GS to detect non-coding or copy number variants that would have been missed by ES was selected because of cost-effectiveness.

It is also important to be aware that NGS serves as an indispensable yet incomplete tool for achieving a definitive diagnosis of IMD. While NGS whether ES/GS, can provide critical information to diagnose IMDs, additional biochemical tests are often needed to confirm the diagnosis. In our group 2 case [8], his UDN ES revealed a known pathogenic variant (c.91C > T (p.R31*)) as well as a VUS (c.454G > A (p.E152K)). Then, respiratory chain functional study was utilized to confirm the diagnosis of complexes II and III deficiency. While our dataset showed that ES/GS was required in 85% of patients to obtain the final diagnosis in the UDN, this case is a good example to highlight the potential pitfall that NGS can be “required but not sufficient” to confirm the diagnosis. Importantly, we need to be aware that NGS can be “sufficient but not always necessary” to confirm the diagnosis. While NGS has high diagnostic yields, NGS is not necessarily diagnostic as a stand-alone application because diagnoses are based on complementary genetic and phenotypic that are synergistic. In our group 1 case [7], the diagnosis of SORD deficiency that was complicated by the paucity of *SORD* genes on commercial neuropathy panels and quantitative sorbitol analysis. It was ultimately solved by combining high quality GS and disease specific metabolite studies. Therefore, while urine and plasma methods for measuring sorbitol levels could have detected SORD deficiency without the need for GS, GS raised the suspicion of SORD deficiency, and this led to a search for a lab to detect sorbitol which was not available until recently. This demonstrates the power of GS to generate diagnostic hypotheses that overcome the limits of commercially available panels, which can then be confirmed with specific metabolic testing. The bias of diagnostic tool availability in the UDN as mentioned above might increase the rate of unnecessary NGS. Considering this potential bias, the actual percentage of UDN subjects whose diagnoses were finalized to have IMDs through ES/GS might be lower than 85%.

Here, we suggest that combining innovative diagnostic approaches as used by the UDN will increase diagnostic yields. However, methods like NGS have potential pitfalls. The utility of not only NGS but also untargeted metabolomic profiling have been compared with traditional screening approaches. The UDN Metabolomics Core offers comprehensive profiling including targeted and untargeted metabolomics approaches, which helped to discover previously unrecognized IMDs [17, 18]. The approach used by Alaimo et al., 2020 to improve the diagnosis of IMD is complementary to our UDN approach [19]. They studied the efficacy of combining integrated untargeted metabolomics and ES results to identify functionally important variants. They found that metabolomic data contributed to the classification of ES variants found in 43.5% of individuals evaluated for IMD studied by both methods. They further demonstrated that comprehensive metabolomic profiling aided in classifying over 90% of the variants and concluded that it could provide functional data to improve ES variant classification. However, they also found that a large number of IMDs were not reliably detected by their approach. These include mitochondrial respiratory complex deficiencies, glycogen storage disorders, most lysosomal storage disorders, and rare enzyme defects not previously assayed by their laboratory. Additionally, in some cases, their findings needed to be confirmed via traditional assays, such as enzyme activity or studies of additional body fluids. This agrees with our need to use advanced metabolic and other techniques to confirm UDN diagnoses that were suggested by NGS results.

Interestingly, Liu et al., compared the efficiency of untargeted metabolomic profiling to traditional screening approach with plasma amino acids, acylcarnitine profiles, and urine organic acids to identify IMD [20]. They found that untargeted metabolomic profiling provided a sixfold higher diagnostic yield compared with the traditional screening approach and identified a broader spectrum of IMD. They noted that, with the expansion of NBS programs, traditional metabolic testing approaches identify few disorders beyond those covered on the NBS. They conclude that their data support the capability of clinical untargeted metabolomic profiling in screening for inborn errors of metabolism and suggest that broader screening approaches should be considered in the initial evaluation for IMDs. This agrees with our finding that advanced metabolic and other techniques were needed to confirm our UDN diagnoses that had been missed by traditional metabolic testing approaches. Our dataset that ES/GS was required in 85% of patients for the final diagnosis in the UDN is conceptually related to the conclusions that Liu et al., and Alaimo et al., reported. Taken together, we conclude combining newer diagnostic approaches including NGS will increase the diagnostic yields over the traditional diagnostic screening approach to diagnose IMD.

In addition to the Metabolomics Core, the UDN also incorporates Model Organism Screening Centers (MOSC). Both can contribute to an understanding of the candidate variants and their associated pathophysiologic roles [21]. The UDN Sequencing Core provides ES/GS and RNA sequencing to improve diagnostic yields in the UDN. Expanding the diagnostic approach beyond NGS will increase diagnostic yields in the UDN, incorporating the Sequencing Core, Metabolomics Core, and MOSC. Additionally, proteomics is an emerging method that can provide additional information on IMDs, including the identification of biomarkers and understanding of the underlying pathophysiology [22].

While our dataset showed NGS increased diagnostic yields, it is still unknown if NGS can be an alternative first-line diagnostic method for IMDs due to some disadvantages. The current NGS, particularly whole genome sequencing, is time-consuming and less sensitive than targeted methods [5]. Also, NGS has some potential disadvantages that include reduced detection of methylation and imprinting variants, repeat expansions, and some copy number variants, and rearrangements [5]. Future work is required to identify potential advantages of long-read sequencing that can overcome many of these pitfalls, ultimately resulting in an increased yield when compared to traditional GS [23]. In addition, as discussed below, NGS is a costly methodology that necessitates specialized facilities, making it less readily available to external patients primarily due to cost and insurance coverage limitations. In theory, shortening the diagnostic odyssey should lead to cost savings on numerous expensive tests. However, a recent scoping review has concluded that there is a profound lack of standardization in recording the diagnostic odyssey [24]. This therefore prevents any effective cost analysis of NGS in the UDN at present. Furthermore, NGS can raise ethical and psychosocial concerns due to potential risks associated with detection of unexpected non-metabolic genetic disorders and generation of many variants of unknown significance (VUS) [25]. Considerable deliberation has been discussed to the potential technical, financial, and ethical dilemmas of the genomic newborn screening [26].

In summary, our UDN data show that the spectrum of IMDs detected in the UDN cohort is large and growing. This was due to the availability through the UDN of more current, robust, and sensitive methods at minimal cost to UDN participants. We hypothesize that the adoption of these and future methods to clinical practice promises to expand the spectrum of known IMDs that will improve diagnosis and treatment that may lead to additional clinical trials. Furthermore, we conclude combinations of multiple diagnostic approaches are required to detect IMDs that are presently missed by the traditional laboratory screening methods.

## Data Availability

Data sharing is not applicable to this article as no new data were created or analyzed in this study.
